# Circulating Irisin Concentrations Are Associated with a Favourable Lipid Profile in the General Population

**DOI:** 10.1371/journal.pone.0154319

**Published:** 2016-04-29

**Authors:** Simon Oelmann, Matthias Nauck, Henry Völzke, Martin Bahls, Nele Friedrich

**Affiliations:** 1 Institute of Clinical Chemistry and Laboratory Medicine, University Medicine Greifswald, Greifswald, Germany; 2 Institute for Community Medicine, University Medicine Greifswald, Greifswald, Germany; 3 Department of Cardiology, University Medicine Greifswald, Greifswald, Germany; 4 German Centre for Cardiovascular Research (DZHK), Partner site Greifswald, Germany; University of the Balearic Islands, SPAIN

## Abstract

**Background/aims:**

Irisin is a myokine, which is mainly inversely associated with the risk for non-communicable diseases. Irisin improves cellular energy metabolism by uncoupling the mitochondrial respiratory chain resulting in increased energy expenditure using lipids. To date potential associations between irisin concentration and lipid profile are poorly understood. Therefore, this investigation aimed to evaluate potential associations between irisin and lipid levels in the general population.

**Methods:**

Data of 430 men and 537 women from the population-based Study of Health in Pomerania (SHIP-TREND) with available irisin and lipid concentrations were used. Analyses of variance, linear and logistic regression models adjusted for age, HBA1c, waist circumference, physical activity, smoking, alcohol consumption, systolic blood pressure, ALAT were calculated.

**Results:**

We detected significantly inverse associations between irisin and circulating levels of total [beta coefficient 0.21 (standard error 0.08), p = 0.01], low-density cholesterol [-0.16 (0.07), p = 0.03] and triglycerides [-0.17 (0.08), p = 0.02] for men. Females without lipid lowering medication had an inverse association between irisin and total cholesterol [-0.12 (0.06), p = 0.05]. Further, male subjects with irisin concentrations in the third tertile had an increased odds for elevated low-density cholesterol [odds ratio 1.96 (95% confidence interval 1.07–3.48), p = 0.03) and triglyceride [1.95 (1.09–3.47), p = 0.02] levels, even after exclusion of subjects with lipid lowering medication. In addition, our data revealed an annual rhythm of serum irisin levels with peak levels arise in winter and summer months.

**Conclusion:**

This is the first investigation to report a significant association between circulating irisin and a favourable lipid profile in the general population. This may infer that higher irisin concentrations are associated with a reduced risk for non-communicable diseases.

## Introduction

Irisin is a metabolite secreted by myocytes and upregulated after moderate aerobic endurance exercise [1]. However, a literature review resulted in conflicting evidence [2]. In detail, while Huh et al. [3] reported an upregulation of irisin concentration after 30 min of acute exercise, Hecksteden et al. [4] did not find any changes in irisin concentrations following 26 weeks of monitored aerobic exercise training. Recently, Jedrychowski et al. [5] found higher irisin levels in subjects participating in aerobic interval training compared to sedentary counterparts. A potential explanation for the conflicting results is that irisin may not be exclusively secreted by skeletal muscle, but also by the perimysium, endomysium and nerve sheaths [6]

As mentioned above, exercise induced irisin release by skeletal muscle has been investigated previously. During physical activity peroxisome proliferator activated receptor gamma coactivator 1 alpha (PGC-1α) expression increases [1, 7] and PGC-1α in turn stimulates fibronectin type 3 domain containing 5 (FDNC5) expression. Following a post-translational proteolytic cleavage of FNDC5, irisin is secreted into extracellular space. While the release of irisin in response to exercise from skeletal muscle is under debate, several physiological functions have been identified. For example, in white adipose tissue (WAT) irisin uncouples the mitochondrial respiratory chain and induces “browning”. Browning of WAT increases basic energy expenditure [7]. Interestingly, type 2 diabetes mellitus (T2DM) is associated with reduced irisin levels [8]. Besides T2DM, irisin is also associated with other metabolic diseases e.g. non-alcoholic fatty liver disease (NAFLD). Interestingly, no linear association between irisin concentration and NAFLD was found but rather an initial increase followed by a decrease with the progression of the disease [9]. Overall, the established role of irisin in whole body homeostasis suggests a therapeutic potential for the treatment of T2DM and obesity [1].

The role of a disturbed lipid profile as a risk factor for various diseases (e.g. cardiovascular diseases) and increased mortality is well established [10]. Currently the relation between irisin and circulating lipids is unclear. Specifically, a positive association between irisin concentration and high-density lipoprotein (HDL) cholesterol was found in patients with chronic kidney disease [11, 12]. A study [3] among middle-aged healthy women detected an inverse association of irisin with HDL and total cholesterol. No significant associations between irisin concentrations and lipid profile were observed in two subgroups of obese Chinese [13, 14]. Unfortunately, previous studies included only a relatively low number of subjects. Further, patient data may not be used to draw conclusions regarding the relation between irisin and circulating lipids in the general population. Therefore, the aim of the present study was to investigate the associations between circulating irisin and lipid levels in a subsample of the population-based Study of Health in Pomerania (SHIP).

## Materials and Methods

### Setting and subjects

SHIP-TREND is the second cohort of the Study of Health in Pomerania (SHIP) a population-based research project in West Pomerania, a rural region in north-east Germany [15]. A stratified random sample of 8,826 adults aged 20–79 years was drawn from population registries. Sample selection was facilitated by centralization of local population registries in the Federal State of Mecklenburg-West Pomerania. Stratification variables were age, sex and city/county of residence. Examinations were conducted between 2008 and 2012. Out of all invitations, 4,420 choose to participate (50.1% response). The study follows the recommendations of the Declaration of Helsinki and was approved by the ethics committee of the University of Greifswald. SHIP data are publically available for scientific and quality control purposes. Data usage can be applied for via www.community-medicine.de. For a subset of 1,001 subjects without self-reported diabetes who underwent an oral glucose tolerance test (OGTT) a more in-depth phenotyping was performed including the measurements of plasma irisin concentrations.

Of these 1,001 subjects, 34 subjects were excluded due to the presence of at least one of the following conditions: estimated glomerular filtration rate <50 mL/min/1.73 m^2^, outliers for irisin (values above the sex-specific 99% percentile) or missing values for confounding factors. The final study population comprised 967 individuals (430 men, 537 women). Furthermore, subgroup analyses were performed after the exclusion of subjects taking lipid lowering medication (men: n = 38; women: n = 34).

### Measurements

Information on age, sex, socio-demographic characteristics, and medical histories were collected by computer-aided personal interviews. Smoking status and physical activity were assessed by self-report. Waist circumference (WC) was measured to the nearest 0.1 cm using an inelastic tape midway between the lower rib margin and the iliac crest in the horizontal plane, with the subject standing comfortably with weight distributed evenly on both feet. The measurement was taken at the level of the narrowest part of the waist. All anthropometric measurements were taken in accordance to World Health Organization standards. After a 5-min resting period, blood pressure was measured three times on the right arm of seated subjects using a digital blood pressure monitor (HEM-705CP, Omron, Tokyo, Japan), with each reading being followed by a further resting period of 3 min. Cuffs were applied according to the circumference of the participant's arm. The mean of the second and third measurements (in mmHg) was used for the analyses.

Fasting blood samples were drawn from the cubital vein in the supine position and aliquots were prepared for immediate analysis and for storage at -80° C. Plasma irisin concentration was measured by a competitive enzyme-linked immunosorbent assay (Adipogen AG, Liestal, Switzerland). The coefficient of variation was 11.57%. Glycated hemoglobin (HbA1c) was measured by high-performance liquid chromatography with spectrophotometric detection (Diamat Analyzer; Bio-Rad, Munich, Germany). Serum alanine aminotransferase (ALAT), total cholesterol, HDL, low-density lipoprotein (LDL) cholesterol and triglycerides were measured using the Dimension Vista 500 analytical system (Siemens AG, Erlangen, Germany). Altered lipid levels were defined based on cut-points found in the third report of the National Cholesterol Education Program Adult Treatment Panel III [16] (total cholesterol, LDL and HDL) or in the guidelines from the World Health Organization (triglycerides) [17]: total cholesterol ≥ 239.75 mg/dl (6.2 mmol/l), LDL cholesterol > 158.55 mg/dl (4.1 mmol/l), triglycerides ≥ 148.75 mg/dl (1.7 mmol/l) and HDL cholesterol < 40.22 mg/dl (1.04 mmol/l). Subjects with elevated total cholesterol, LDL cholesterol, reduced HDL cholesterol or the self-reported use of lipid-lowering drugs (anatomical-therapeutic-chemical (ATC) code C10) were considered to be dyslipidemic.

### Statistical analysis

**Fig 1:**
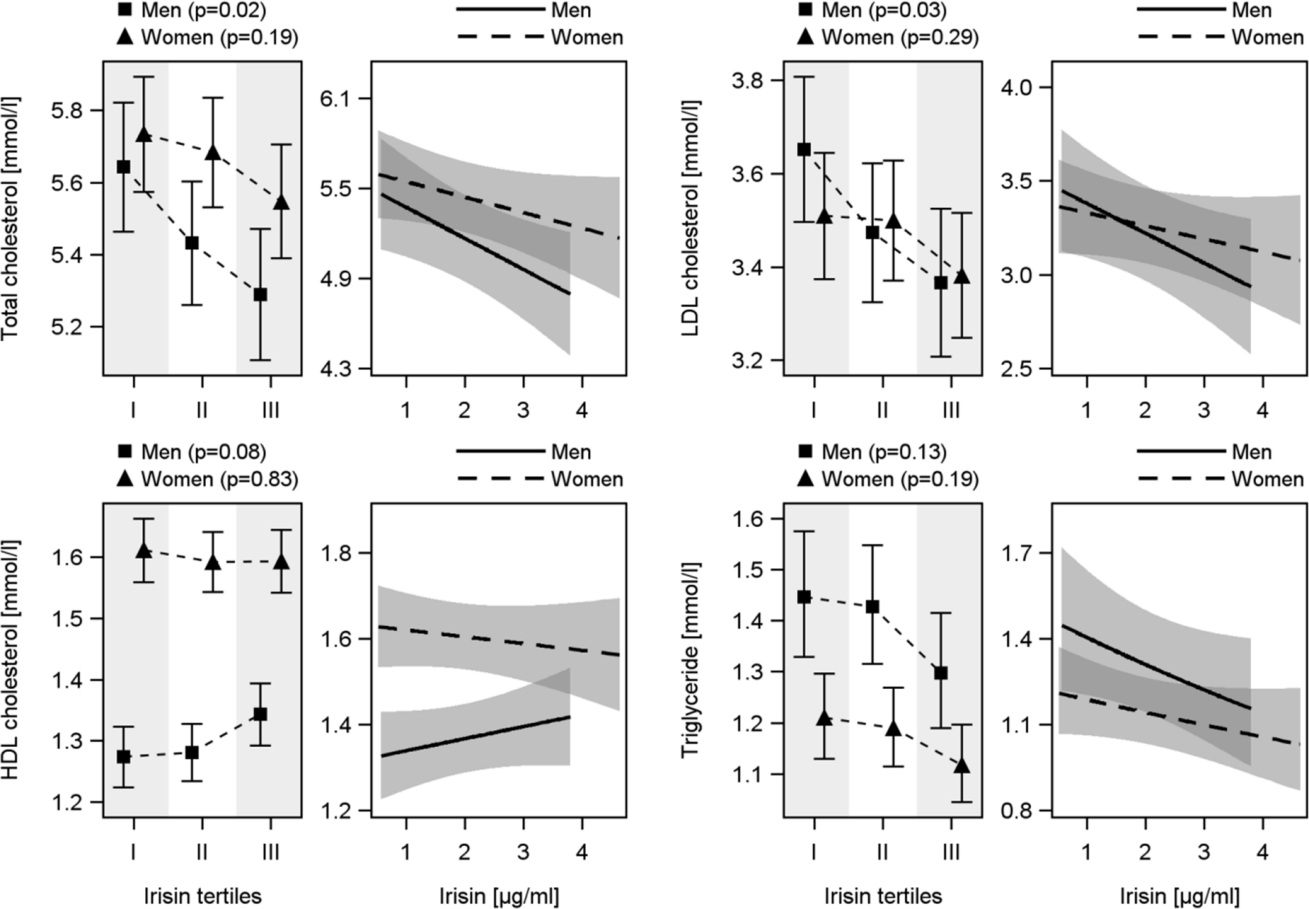
Association between irisin concentrations and levels of total cholesterol, low-density lipoprotein (LDL), high-density lipoprotein (HDL) cholesterol and triglyceride among men and women. *Left side of each lipid*: Estimated mean levels with 95% confidence interval by tertiles of irisin. *Right side of each lipid*: Linear regression. All models were adjusted for age, HBA1c, waist circumference, physical activity, smoking, alcohol consumption, systolic blood pressure, ALAT and months of examination (only linear regression).

Continuous data were expressed as median (25^th^; 75^th^ quartile). Nominal data were expressed as percentage. For bivariate analyses the Kruskal-Wallis test (continuous data) or χ^2^-test (nominal data) were used to compare men and women. Sex-specific analysis of variance (ANOVA) was carried out to calculate adjusted means for lipid parameters in groups of irisin [due to the found annual rhythm (Fig 1) categorisation according to sex- and months-specific tertiles]. Multivariable linear regression models were separately performed in men and women to estimate independent associations of irisin as a continuous variable with total, LDL, HDL cholesterol, and triglycerides. For all analyses, to detect possible nonlinear associations, models with restricted cubic splines with 3 knots pre-specified located at the 5th, 50th, and 95th percentile as recommended by Stone and Koo [18] were compared by likelihood ratio test to the fit of linear model. No nonlinear relations were detected resulting in linear models. The full models were adjusted for age, WC, HbA1c, ALAT, smoking, physical activity, alcohol consumption and systolic blood pressure. All analyses were performed for the whole population as well as after exclusion of subjects taking lipid lowering medication (men: n = 38; women: n = 34). A value of p < 0.05 was considered statistically significant. Statistical analyses were performed with SAS 9.4 (SAS Institute Inc., Cary, NC, USA).

## Results

The baseline characteristics of men and women are described in Table 1. Men were more often smokers or former smokers, consumed more alcohol and had higher WC, HbA1c levels and systolic blood pressure than women. Furthermore, men had lower total cholesterol and HDL cholesterol but higher triglyceride levels compared to women. Compared to non-participants, participating men and women were younger, more physically active, had a lower WC, HbA1c as well as triglyceride levels and were less affected by hypertension. Additionally, men had higher LDL and HDL cholesterol levels, whereas in women no significant differences for these parameters became apparent (data not shown). With respect to irisin levels no sex and age difference were observed (Table 1 and S1 Fig).

**Table 1:** Baseline Characteristics stratified by sex.

Characteristics	Men (n = 430)	Women (n = 537)	p^a^
Age (years)	50 (40; 61)	50 (40; 60)	0.91
Smoking (%)			<0.01
never smokers	32.3	50.1	
former smokers	44.7	28.9	
current smokers	23.0	21.2	
Physically active (%)	73.0	73.7	0.80
Alcohol consumption (g/day)	8.6 (3.1; 18.4)	2.4 (0.7; 5.5)	<0.01
Waist circumference (cm)	94 (87; 102)	81 (74; 90)	<0.01
Systolic blood pressure (mmHG)	131 (121; 140)	117 (108; 129)	<0.01
Hypertension (%)	43.5	34.3	<0.01
ALAT (μkatal/l)	0.46 (0.35; 0.64)	0.30 (0.24; 0.42)	<0.01
HbA1c (%)	5.2 (4.9; 5.5)	5.1 (4.8; 5.5)	<0.01
Total cholesterol (mg/dl)	5.3 (4.6; 6.1)	5.5 (4.9; 6.3)	<0.01
LDL cholesterol (mg/dl)	3.4 (2.8; 4.0)	3.3 (2.7; 4.0)	0.35
HDL cholesterol (mg/dl)	1.27 (1.10; 1.47)	1.58 (1.34; 1.83)	<0.01
Triglyceride (mg/dl)	1.32 (0.92; 1.92)	1.16 (0.84; 1.61)	<0.01
Irisin (μg/ml)	1.89 (1.46; 2.58)	1.97 (1.48; 2.67)	0.40

LDL = low-density lipoprotein; HDL = high-density lipoprotein; ALAT = alanine aminotransferase. Continuous data are expressed as median (25^th^ percentile; 75^th^ percentile); nominal data are given as percentages.

^a^χ^2^-test (nominal data: smoking, physical activity, diabetes) or Mann-Whitney test (interval data: remaining comparisons) were performed. A value of p < 0.05 was considered statistically significant.

A significantly inverse association between irisin concentration and total as well as LDL cholesterol was identified for male subjects. In detail, total and LDL cholesterol decreased by 0.36 mmol/l and 0.28 mmol/l from the lowest to the highest irisin tertile, respectively. Furthermore, a trend towards a positive association with HDL cholesterol became apparent in men (Fig 1). All findings were confirmed after the exclusion of men taking lipid lowering medication (S2 Fig) even if some relations were short of being statistically significant. In women, no significant association between irisin and lipid parameters were found (Fig 1 and S2 Fig).

**Fig 2:**
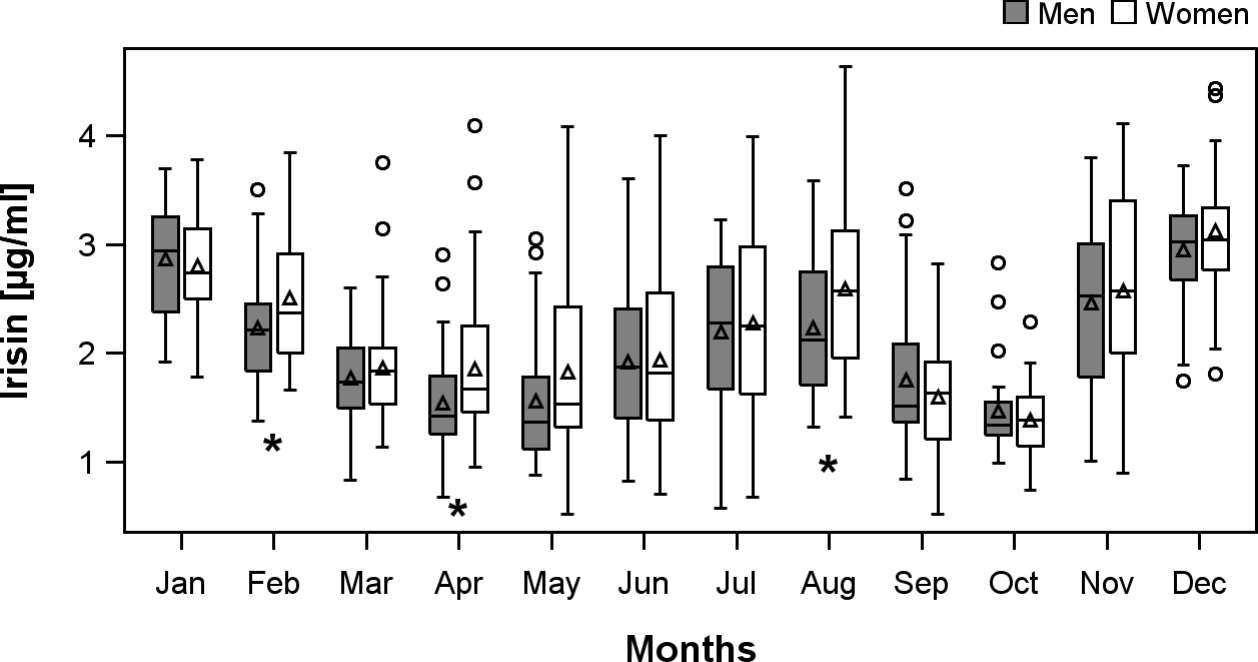
Boxplots of irisin concentrations separately for men and women by month. *p<0.05 for comparison between men and women. The black triangles indicate group means.

Linear regression analyses confirmed inverse associations of irisin with total and LDL cholesterol in men even after exclusion of those taking lipid lowering medication (Fig 2 and S2 Fig, Table 2). Moreover, a significantly inverse relation with triglycerides became apparent for male subjects. Among women without lipid lowering medication a significantly inverse relation between irisin and total cholesterol was found (S2 Fig, Table 2). No other significant associations could be detected for LDL, HDL cholesterol and triglyceride in women.

**Table 2:** Association between irisin concentrations and lipid levels.

	Total cholesterol		LDL cholesterol		HDL cholesterol		Triglyceride	
	beta (StdErr)	p	beta (StdErr)	p	beta (StdErr)	p	beta (StdErr)	p
**All subjects (n = 967)**								
Women	-0.103 (0.062)	0.10	-0.070 (0.053)	0.19	-0.016 (0.020)	0.44	-0.059 (0.038)	0.12
Men	-0.207 (0.083)	0.01	-0.159 (0.073)	0.03	0.028 (0.023)	0.22	-0.173 (0.076)	0.02
**Subjects without lipid lowering medication (n = 595)**								
Women	-0.124 (0.064)	0.05	-0.087 (0.054)	0.11	-0.014 (0.021)	0.52	-0.064 (0.040)	0.11
Men	-0.179 (0.083)	0.03	-0.122 (0.073)	0.09	0.015 (0.024)	0.54	-0.145 (0.077)	0.06

LDL = low-density lipoprotein; HDL = high-density lipoprotein; OR = odds ratio; CI = confidence interval; StdErr = standard error.

*Models were adjusted for age, HBA1c, waist circumference, physical activity, smoking, alcohol consumption, systolic blood pressure, ALAT and months of examination. A value of p < 0.05 was considered statistically significant.

With respect to altered lipid levels, logistic regression models (Table 3) revealed that men with irisin concentrations in the lowest tertile had higher odds of elevated LDL cholesterol and triglycerides compared to men with irisin levels in the highest tertile. After exclusion of men taking lipid lowering medication, marginally significantly increased odds of dyslipidemia among men with low irisin levels became apparent (S1 Table). In women, low irisin concentrations were related to higher odds of elevated total cholesterol and dyslipidemia. After exclusion of women taking lipid lowering medication the main results were confirmed (S1 Table).

**Table 3:** Association between irisin concentrations and lipid levels as well as dyslipidemia.

	Elevated total cholesterol		Elevated LDL cholesterol		Reduced HDL cholesterol		Elevated triglyceride		dyslipidemia	
	OR (95%-CI)	p	OR (95%-CI)	P	OR (95%-CI)	P	OR (95%-CI)	P	OR (95%-CI)	p
**Men**										
N (cases)	430 (104)		430 (99)		430 (79)		430 (142)		430 (215)	
Irisin per unit decrease	1.33 (0.89; 2.00)	0.17	1.50 (0.99; 2.28)	0.06	0.99 (0.63; 1.57)	0.98	1.61 (1.06; 2.44)	0.03	1.25 (0.87; 1.79)	0.23
Irisin (ref: III tertile)										
II tertile	1.24 (0.70; 2.20)	0.45	1.18 (0.65; 2.12)	0.59	1.40 (0.74; 2.63)	0.30	1.12 (0.64; 1.96)	0.68	1.08 (0.66; 1.77)	0.76
I tertile	1.60 (0.89; 2.87)	0.11	1.93 (1.07; 3.48)	0.03	0.93 (0.47; 1.86)	0.84	1.95 (1.09; 3.47)	0.02	1.41 (0.84; 2.38)	0.20
**Women**										
N (cases)	537 (152)		537 (119)		537 (13)		537 (116)		537 (204)	
Irisin per unit decrease	1.35 (0.99; 1.84)	0.06	1.32 (0.94; 1.85)	0.11	-	-	1.14 (0.82; 1.58)	0.44	1.35 (1.00; 1.82)	0.05
Irisin (ref: III tertile)										
II tertile	1.78 (1.08; 2.93)	0.02	1.40 (0.81; 2.41)	0.23	-	-	1.30 (0.74; 2.27)	0.36	1.81 (1.12; 2.93)	0.02
I tertile	1.54 (0.94; 2.54)	0.09	1.46 (0.86; 2.50)	0.16	-	-	1.43 (0.83; 2.47)	0.20	1.59 (0.99; 2.58)	0.06

LDL = low-density lipoprotein; HDL = high-density lipoprotein; OR = odds ratio; CI = confidence interval. Models were adjusted for age, HBA1c, waist circumference, physical activity, smoking, alcohol consumption, systolic blood pressure, ALAT and months of examination. A value of p < 0.05 was considered statistically significant.

Additionally, we found an annual rhythm of irisin concentrations. Irisin had peak levels in winter (December–February) and summer (July-August) while during spring (April-May) and fall (September-October) lower levels of irisin were measured (Fig 2). Men and woman showed similar rhythms. Nevertheless, men had non-significantly lower irisin concentrations in most months compared to women.

## Discussion

In the present study we investigated the association between irisin concentrations with circulating lipid levels in a subsample of the population based SHIP-TREND cohort. We found inverse associations between irisin concentrations and total, LDL cholesterol as well as triglycerides among women or men even after exclusion of subjects taking lipid lowering medication. The associations in women were mostly weaker resulting in lower and partly non-significant estimates compared to men. However, the direction of the detected associations remained the same. Furthermore, we detected higher odds of elevated lipid levels in subjects with low irisin concentrations. Therefore, this is the first investigation to report a significant association between irisin concentrations and a beneficial lipid profile in a large population-based study.

We observed significant differences in metabolic risk profile between males and females. Specifically, males had higher blood pressure, triglyceride levels, consumed more alcohol and had lower HDL cholesterol levels compared to women. The resulting lower cardiometabolic risk in women may partly explain the observed lower association between irisin and blood lipids. Previous studies investigating the associations of irisin with metabolic outcome including insulin or the Homeostatic Model Assessment for Insulin Resistance (HOMA-IR) [19] as well exercise related outcomes [20, 21] also reported significant associations in men but not women. The authors discussed sexual dimorphism in body composition with a higher lean and lower fat mass in men as well as differences in transcription of FNDC5 or sex hormones levels as possible reason for the divergent findings between men and women. However, even though the relations between irisin and lipid levels were partly non-significant in women, the directions of associations were similar for males and females. Therefore, our results may also be explained by a lack of statistical power. The number of women may have been too low to detect the weaker associations in women and thus even larger studies are needed.

After its discovery in 2012 irisin was given a great therapeutic potential for the treatment of obesity, metabolic syndrome and non-communicable disease [1]. The expected positive impact of irisin was based on the irisin-related “browning” of WAT or beige adipose tissue into brown adipose tissue [7]. Irisin increases the expression of mitochondrial uncoupling protein 1 (UCP1). An upregulation of UCP1 increases total energy expenditure and energy dissipation by uncoupling the respiratory chain. Initially irisin was thought to be secreted after moderate endurance exercise [1]. Even though confirmed by several studies, another investigation found no association between irisin secretion and exercise [2]. Beside the exercise related effect of irisin, during the last years several studies were performed to examine further metabolic effects of irisin. In 2014 Panagiotou et al. [22] discussed the potential of irisin playing a role in lipid metabolism and lipoprotein sub-particle regulation. In agreement with this, higher levels of irisin were found in patients taking simvastatin as lipid lowering medication [23]. However, the number of investigations exploring associations of irisin concentrations with lipid levels is still limited and the results are conflicting as discussed below [3, 11–14, 22–25].

In line with our findings of an inverse association between irisin and total cholesterol, a study [23] among 72 male subjects with mild hypercholesterolemia and an investigation [3] among 117 middle-aged healthy women reported an inverse relation of irisin with total cholesterol. Furthermore, the latter study [3] also showed a trend towards an inverse correlation with LDL cholesterol and unexpectedly HDL cholesterol. The inverse association between irisin and HDL cholesterol was also found in 39 older subjects with either diabetes or two other cardiovascular risk factors [22]. In the present study, even though we found inverse associations to total, LDL cholesterol and triglycerides, we did not observe an association with HDL cholesterol in either men or women. However, in agreement with our determination of a favourable lipid profile in subjects with high irisin concentrations, among patients with chronic kidney disease irisin levels were independently positively correlated with HDL cholesterol [11, 12]. Therefore, several studies were in concordance with our findings.

In contrast to our findings, other studies reported no significant associations between irisin and lipid levels [13, 14] as well as positive relations with total, LDL cholesterol or triglycerides among non-diabetic subjects [24] or subjects suffering from kidney disease [11]. Furthermore, among 151 Caucasian and African American subjects, high irisin concentrations were related to increased odds of high triglyceride. High irisin concentrations were associated with decreased odds HDL cholesterol levels [25] as well as high odds of LDL cholesterol in obese Chinese [26].

In conclusion, the previously conducted investigations show an inconsistent picture regarding the association of irisin with the lipid profile. One possible reason for the partially conflicting findings might be relatively low number of study participants ranging between 39 and 151 [3, 12, 22–25]. Even in the present study population we observed a borderline significant association between irisin and lipid profile in over 500 women arguing for the use of larger study population. Furthermore, previous studies assessed irisin concentration in patients with hypercholesterolemia [3] or chronic kidney disease [11, 12]. These primary diseases might directly interfere with the investigated association or indirectly via altered risk factors or confounders of the exposure or outcome. Therefore, their data cannot be easily transferred to a general population. In general, further research is needed to provide comprehensive understanding of irisin related physiological effects and possible implications in clinical implications.

Other important results in the present study population are 1) the age-independency of irisin concentrations and 2) the annual rhythm of irisin which we previously detected [20]. Up to now, there is conflicting evidence regarding a potential relation between irisin concentrations and age. While several groups [3, 4, 11, 27, 28] reported an inverse correlation another investigation [24] revealed the opposite of a positive correlation. However, also in agreement with our results, no significant relation between irisin and age was previously observed [23, 24, 29]. Specifically, conflicting results for T2DM subjects were found with observed inverse [27], positive [29] as well as no [24] relation to age. Overall, the relation between age and irisin is not fully clarified even if the present large study argues for no age-dependency of irisin concentrations. Regarding the annual rhythm two peaks, one during the summer and the other during winter months, were found in to our data. A possible explanation for the winter peak could be the overall increased energy expenditure for thermogenesis to compensate cool temperatures. The findings of Paul Lee et al. [30] could confirm this hypothesis. They detected higher concentrations of irisin in shivering subjects potentially compensating for lower temperatures. A possible reason for the summer peak was described by Aydin and colleagues [31]. They reported increased irisin concentrations in subjects taking a hot Turkish bath over 45 minutes. We hypothesize that heat in general may be a stimulus for irisin secretion. This annual rhythm of irisin concentrations may be another explanation for the conflicting results that exist about irisin. Further research is needed to clarify the annual rhythm of irisin concentrations.

Strengths of our study are the large sample size and the precise assessment of confounding factors. After excluding subjects taking lipid lowering medication our main results were confirmed. However, there are also limitations. The current study population represents a subsample of the whole SHIP-TREND population whereby the investigated subjects participated in an OGTT. Due to the fact that these subjects have to be free of diabetes, participants were younger and healthier than non-participants. Therefore, the generalisation of our results might be limited. There might be further confounding factors which are currently unknown. Further studies are needed to discover the exact biochemical pathways and therapeutic potential of irisin. Another limitation may arise from missing SI units for the ELISA Kit. Furthermore, Albrecht and colleagues [32] recently questioned the quality of different ELISA kits for the measurement of irisin concentrations. They described cross-reactions with non-specific serum proteins that influence the accuracy of ELISA kits. But Polyzos et al. [33] showed that several kits have been proven to validly detect irisin levels. Furthermore, a recent study [5] confirmed the existence of irisin in human plasma by quantitative mass spectrometry and its link to physical activity. Nevertheless, further studies are results needed to confirm the involvement of irisin in lipid metabolism.

In conclusion, our study is the first to report sex-specific associations between irisin concentrations and circulating lipids in the general population. Our data showed an improved lipid profile in association with irisin. One may speculate that the positive association may infer an irisin-induced protection against lipid-related non-communicable disease. However, further study about irisin and its biochemical pathways as well as the accurate function in humans is needed.

## Supporting Information

S1 Fig Irisin concentrations separately for men and women by age. *Left side*: Scatterplot of irisin levels versus age by sex. *Right side*: Boxplots showing 25th, 50th and 75th percentiles (horizontal bars), and 1.5 interquartile ranges (error bars) of irisin levels for different age groups separately for men and women.
(PDF)

S2 Fig Association between irisin concentrations and levels of total cholesterol, low-density lipoprotein (LDL), high-density lipoprotein (HDL) cholesterol and triglyceride among men and women without taking lipid lowering medication. *Left side of each lipid*: Estimated mean levels with 95% confidence interval by tertiles of irisin. *Right side of each lipid*: Linear regression. All models were adjusted for age, sex, HBA1c, waist circumference, physical activity, smoking, alcohol consumption, systolic blood pressure, ALAT and months of examination (only linear regression).
(PDF)

S1 Table Association between irisin levels and lipid levels as well as dyslipidemia in subjects without lipid medication. LDL = low-density lipoprotein; HDL = high-density lipoprotein; OR = odds ratio; CI = confidence interval. Models were adjusted for age, sex, HBA1c, waist circumference, physical activity, smoking, alcohol consumption, systolic blood pressure, ALAT and months of examination.
(PDF)
