# Supplementary figures and images for: Circulating Irisin Concentrations Are Associated with a Favourable Lipid Profile in the General Population
Source: PLoS One. 2016 Apr 29;11(4):e0154319. doi: 10.1371/journal.pone.0154319 (PMC4851367; doi:10.1371/journal.pone.0154319)

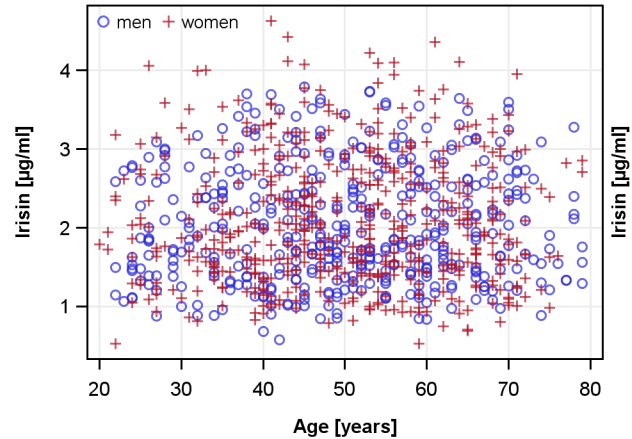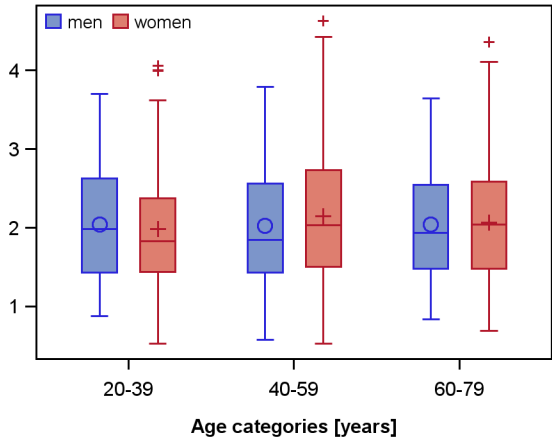

Supplement: S1 Fig — Left side: Scatterplot of irisin levels versus age by sex. Right side: Boxplots showing 25th, 50th and 75th percentiles (horizontal bars), and 1.5 interquartile ranges (error bars) of irisin levels for different age groups separately for men and women. (PDF) [file pone.0154319.s001.pdf]
