# Supplementary material for: Circulating Irisin Concentrations Are Associated with a Favourable Lipid Profile in the General Population
Source: PLoS One. 2016 Apr 29;11(4):e0154319. doi: 10.1371/journal.pone.0154319 (PMC4851367; doi:10.1371/journal.pone.0154319)
Supplement: S2 Fig — Left side of each lipid: Estimated mean levels with 95% confidence interval by tertiles of irisin. Right side of each lipid: Linear regression. All models were adjusted for age, sex, HBA1c, waist circumference, physical activity, smoking, alcohol consumption, systolic blood pressure, ALAT and months of examination (only linear regression). (PDF) [file pone.0154319.s002.pdf]

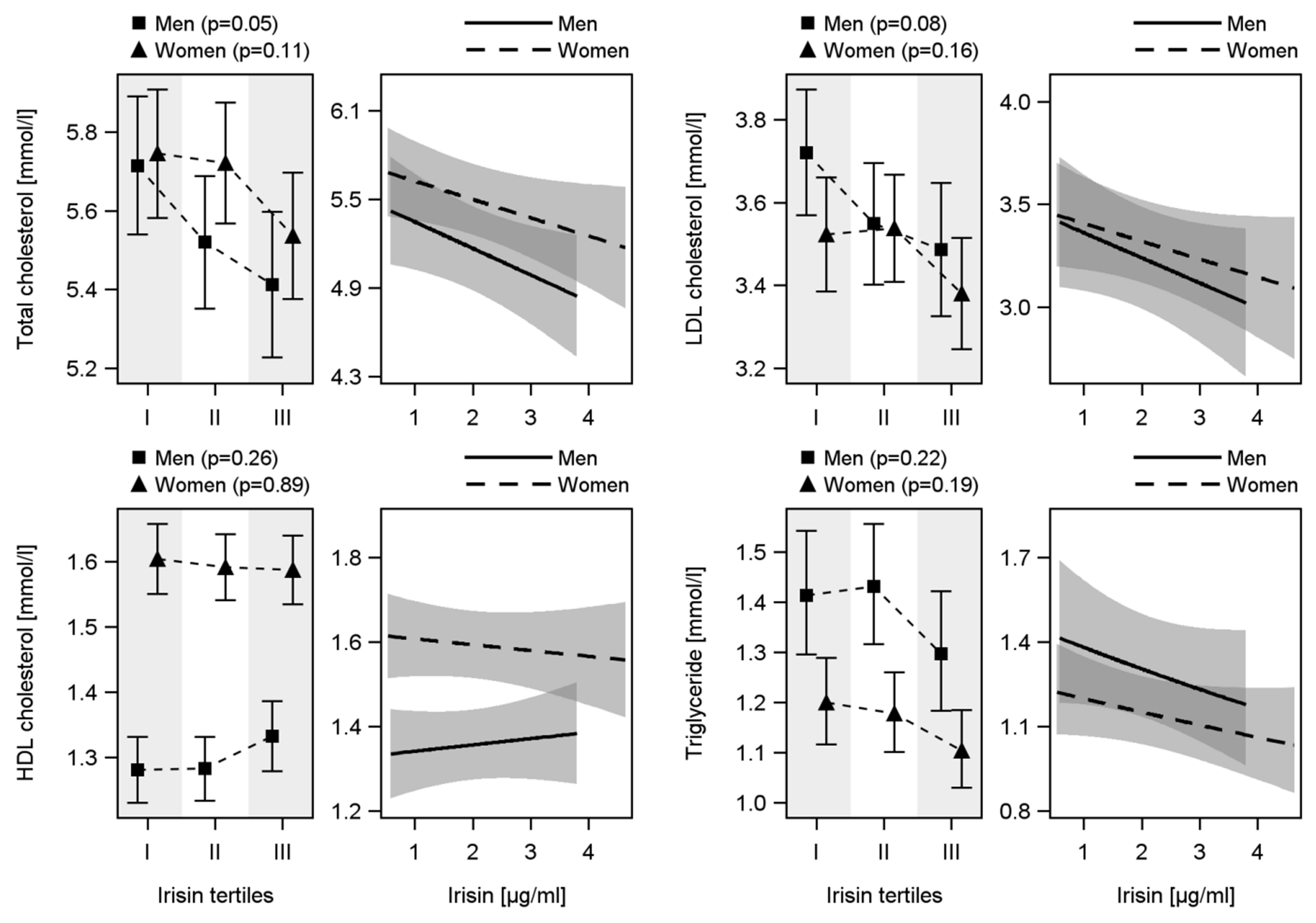

Men (p=0.05)
Women (p=0.11)
Men
Women
Total cholesterol [mmol/l]
Men (p=0.08)
Women (p=0.16)
Men
Women
LDL cholesterol [mmol/l]
Men (p=0.26)
Women (p=0.89)
Men
Women
HDL cholesterol [mmol/l]
Men (p=0.22)
Women (p=0.19)
Men
Women
Triglyceride [mmol/l]
Irisin tertiles
Irisin [µg/ml]
Irisin tertiles
Irisin [µg/ml]
