# Supplementary material for: Circulating Irisin Concentrations Are Associated with a Favourable Lipid Profile in the General Population
Source: PLoS One. 2016 Apr 29;11(4):e0154319. doi: 10.1371/journal.pone.0154319 (PMC4851367; doi:10.1371/journal.pone.0154319)
Supplement: S1 Table — LDL = low-density lipoprotein; HDL = high-density lipoprotein; OR = odds ratio; CI = confidence interval. Models were adjusted for age, sex, HBA1c, waist circumference, physical activity, smoking, alcohol consumption, systolic blood pressure, ALAT and months of examination. (PDF) [file pone.0154319.s003.pdf]

Table S1. Association between irisin levels and lipid levels as well as dyslipidemia in subjects without lipid medication.

	Elevated total cholesterol		Elevated LDL cholesterol		Reduced HDL cholesterol		Elevated triglyceride		dyslipidemia	
	**OR (95%-CI)**	**p**	**OR (95%-CI)**	**P**	**OR (95%-CI)**	**P**	**OR (95%-CI)**	**P**	**OR (95%-CI)**	**p**
**Men**										
N (cases)	392 (103)		392 (98)		392 (71)		392 (127)		392 (177)	
Irisin per unit decrease	1.39 (0.91; 2.12)	0.13	1.54 (1.00; 2.38)	0.05	0.98 (0.60; 1.61)	0.94	1.49 (0.96; 2.32)	0.08	1.41 (0.95; 2.07)	0.08
Irisin (ref: III tertile)										
II tertile	1.19 (0.66; 2.14)	0.56	1.14 (0.62; 2.08)	0.68	1.52 (0.78; 2.98)	0.22	1.16 (0.64; 2.11)	0.62	1.18 (0.70; 1.99)	0.54
I tertile	1.56 (0.86; 2.85)	0.15	1.91 (1.04; 3.50)	0.04	0.96 (0.46; 2.01)	0.92	1.92 (1.04; 3.56)	0.04	1.58 (0.92; 2.74)	0.10
**Women**										
N (cases)	503 (148)		503 (115)		503 (13)		503 (109)		503 (170)	
Irisin per unit decrease	1.40 (1.01; 1.94)	0.04	1.34 (0.95; 1.91)	0.10	-	-	1.21 (0.85; 1.71)	0.30	1.32 (0.97; 1.80)	0.07
Irisin (ref: III tertile)										
II tertile	1.89 (1.13; 3.16)	0.02	1.47 (0.84; 2.57)	0.18	-	-	1.25 (0.70; 2.24)	0.45	1.81 (1.10; 2.97)	0.02
I tertile	1.56 (0.93; 2.62)	0.09	1.44 (0.82; 2.50)	0.20	-	-	1.54 (0.87; 2.72)	0.13	1.58 (0.96; 2.61)	0.07

LDL = low-density lipoprotein; HDL = high-density lipoprotein; OR = odds ratio; CI = confidence interval. Models were adjusted for age, sex, HBA1c, waist circumference, physical activity, smoking, alcohol consumption, systolic blood pressure, ALAT and months of examination.
